# Anterior and Mid-Penile Hypospadias Repair with TIP Technique—Is It Possible with 20-Hour Catheterization?

**DOI:** 10.3390/diseases12110279

**Published:** 2024-11-05

**Authors:** Vasileios Tatanis, Paraskevi Katsakiori, Theodoros Spinos, Angelis Peteinaris, Spyridon Polyzonis, Theofanis Vrettos, Panagiotis Kallidonis, Evangelos Liatsikos, George Zoupanos

**Affiliations:** 1 Department of Urology, University of Patras, 26504 Patras, Greece; tatanisbas@gmail.com (V.T.); vkatsak@gmail.com (P.K.); thspinos@otenet.gr (T.S.); peteinarisaggelis@gmail.com (A.P.); spyrpolyzonis@gmail.com (S.P.); pkallidonis@yahoo.com (P.K.); 2 Department of Anesthesiology and ICU, University of Patras, 26504 Patras, Greece; teovret@gmail.com; 3 Department of Urology, Medical University of Vienna, 1090 Vienna, Austria; 4 Department of Pediatric Urology, Medical Center of Athens, 15125 Athens, Greece; info@zoypanos.gr

**Keywords:** hypospadias, hypospadias repair, TIP urethroplasty, 20-h catheterization, early catheter removal

## Abstract

Background/Objectives: Several surgical procedures have been proposed for the treatment of hypospadias, a common and heterogeneous congenital abnormality in males. Most surgeons utilize either a urethral stent or bladder catheter for 2–14 days as a postoperative urine diversion method depending on the severity of each case and the surgeon’s preferences. The aim of the present study was to evaluate the feasibility of anterior and mid-penile hypospadias repair while placing the urethral catheter for only 20 h. Methods: A single-centre retrospective study was conducted, including all patients who underwent anterior or penile hypospadias repair from January 2012 to January 2023. All operations were performed based on the Tubularized Incised Plate (TIP) Urethroplasty. At the end of the operation, a 6 Fr 100% silicon catheter was stabilized. The catheter was removed 20 h postoperatively, and all patients were discharged on the first postoperative day. The primary endpoint of the study was the complication rate. Results: In total, 104 patients were enrolled. Eighty (76.9%) patients presented with distal penile hypospadias, while 24 patients (23.1%) had mid-penile hypospadias. The mean age at the operation was 20.8 ± 12.4 months, while the mean operative time was 114.2 ± 28.7 min. All the operations were successfully completed. During the follow-up period (mean duration 101.1 ± 44.8 months), complications were recorded in 16.3% of the patients. Conclusions: The application of 20-h catheterization seems to be an effective alternative with outcomes comparable to other conventional drainage approaches, as it reduces the discomfort of the patients without increased risk of complications.

## 1. Introduction

Hypospadias constitutes a common congenital abnormality in males with an approximate incidence of 18.6 in 10,000 live births in Europe [1]. It is characterized by a ventrally ectopic urethral meatus, a ventral penile curvature, and a dorsally hooded foreskin [1,2]. The present literature incriminates several factors such as genetic, hormonal, and environmental in the etiology of this defect [1]. The potential complications during its surgical treatment, as well as the long-term aesthetic, functional, and psychosexual burden to the patient, have given rise to several studies on the best treatment approach in hypospadias patients. The primary objectives of hypospadias repair are the reconstruction of the penile urethra, the transfer of the urethral meatus to the top of the glans, and the correction of the potential penile curvature. All the aforementioned aims should be achieved under the restriction of the postoperative discomfort and complication risk while ameliorating both aesthetic and functional surgical results [3]. Incorrect surgical techniques can cause most of the frequent early postoperative problems like edema, hematoma development, wound dehiscence, and fistula formation [4]. In this direction, several surgical procedures have been proposed for successful hypospadias repair [4,5,6]. However, 25% of the patients still require a second procedure [1].

The management of postoperative urine output constitutes a topic of controversy among the numerous surgical techniques. Most surgeons utilize either a urethral stent or bladder catheter for 2–14 days as a postoperative urine diversion method depending on the severity of each case [7]. Because of the low level of evidence, neither the European Association of Urology (EAU) nor the European Society of Pediatric Urology (ESPU) concludes with specific guidelines concerning the type or duration of post-operative stenting or catheter drainage [8]. Despite the use of analgetic and anticholinergic treatment, the discomfort caused by the urethral catheter constitutes a major concern in hypospadias repair. The removal of the catheter immediately after surgery may diminish the discomfort of both the patient and his relatives leading to early discharge, whereas the early removal of the catheter may increase the risk of urethrocutaneous fistula occurrence as well as wound dehiscence due to the urine flow across the suture. On the contrary, the risk of wound infection, meatal stenosis, and urethral stricture may be increased in case of prolonged catheter drainage. Recently published literature has reported the safety and efficacy of catheter-less urethroplasty [9,10].

The aim of the present study was to evaluate the feasibility of anterior and mid-penile hypospadias repair while placing the urethral catheter for only 20 h.

## 2. Materials and Methods

A retrospective study was conducted including patients—irrespective of their age—who underwent surgical repair for anterior or mid-penile hypospadias by a single surgeon from January 2009 to January 2023 in a single institution. The exclusion criteria included the presence of proximal or penoscrotal hypospadias, the performance of secondary hypospadias repair, severe chordee, the history of neuromuscular diseases or injury at the genitalia, the application of cortisone for any reason, and patients who were lost during follow-up. All patients underwent detailed clinical examination and routine laboratory tests (complete blood count and biochemical tests) preoperatively based on the anesthesiologists’ recommendations. During the examination of the penis, the presence of a chordee and the width of the glans were recorded. In the case of glandular width less than 14 mm, two doses of intramuscular testosterone enanthate (2 mg/kg) were administered eight and four weeks preoperatively. Informed consent for the participation in the study was obtained from the parents/legal guardians.

### 2.1. Surgery Technique and Intraoperative Data

**Figure 1:**
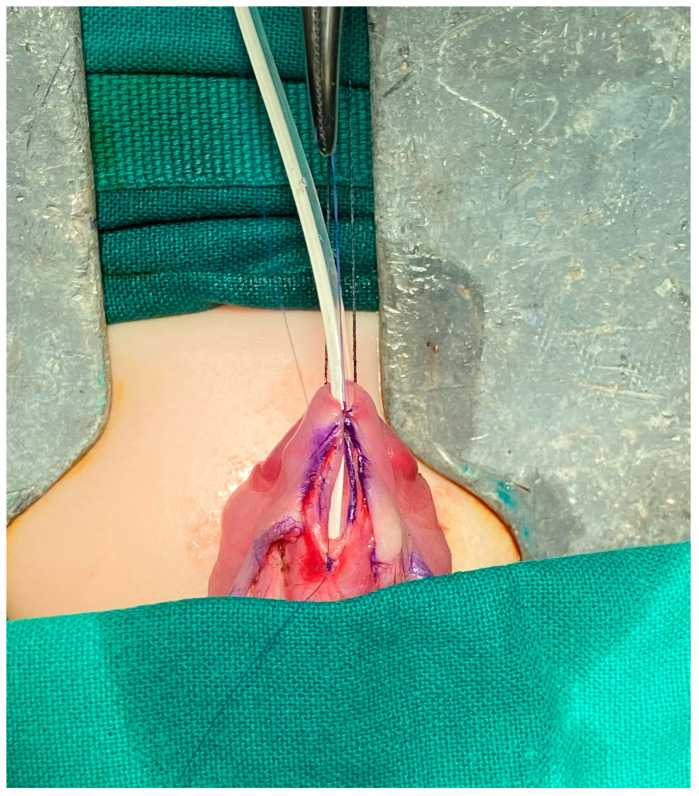
The tubularization of the urethral plate.

**Figure 2:**
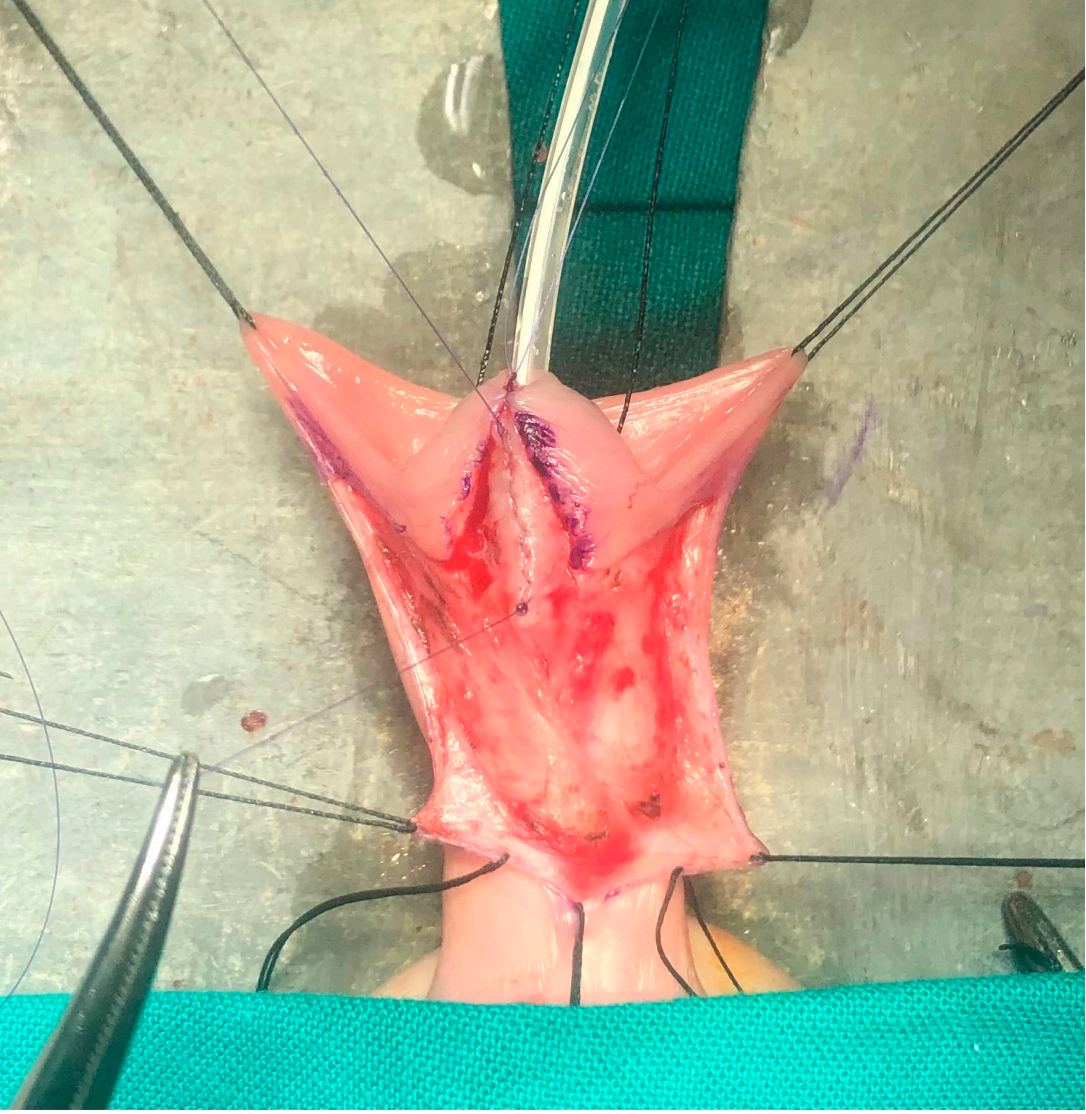
The watertight urethral suture.

All operations were performed by a single surgical team using the Tubularized Incised Plate (TIP) Urethroplasty technique under general anesthesia (Figure 1) [11]. Optical loupe magnification was utilized during all surgeries. The penile urethral plate was tubularized into two layers using a 6-0 PDS continuous watertight extramucosal suture line (Figure 2). The glandular urethral plate was also tubularized in two layers, and the glans in one layer using a 6-0 PDS watertight suture. A tourniquet was not utilized as 1% lidocaine diluted with 1:200,000 epinephrine was applied in the glans intraoperatively [12]. The prepuce was reconstructed based on the Righini technique [13], while in the case of preputial stenosis, the Duhamel technique was applied [14]. A 6 Fr Foley 100% silicon catheter was placed and stabilized in each patient. Based on the age of the patient, the catheter drained into a double-diaper or urine bag. In all cases, an intraoperative weight-adjusted single dose of cefuroxime was administrated.

### 2.2. Postoperative Data

Postoperative management included the administration of weight-adjusted doses of cephalosporins and oxybutynin for relieving bladder spasms in all patients. Additional oral analgetic agents, including regular paracetamol and non-steroidal anti-inflammatory agents for severe pain, were administered. Nutrition was started 3–4 h postoperatively in all patients. The urethral catheter was removed 20 h postoperatively, and the patients were discharged after successful urination. The 20-h catheterization was selected as it may provide the advantages of preventing early complications due to urinary retention caused by postoperative edema and diminishing the discomfort of patients, as there is no need for additional analgesics and spasmolytics.

All patients were followed up one month postoperatively and urethral calibration with an 8 FR bougie was performed. Afterward, the patients were followed up in 2.5 months for the retraction of the foreskin, as earlier retraction may lead to dehiscence, and at 12 months postoperatively. Until the final follow-up, which was performed at the age of 15 years, the patients were examined in case of any symptoms. In case of urination symptoms or at the last follow-up, the patients underwent uroflowmetry. During the follow-up, the evaluation of meatal stenosis, fistula or diverticulum formation, and partial or complete glandular dehiscence were recorded. Meatal or urethral stenosis was diagnosed when the flow rate was less than 5 mL/sec and the plateau pattern was present. Surgery success was defined as the presence of an anatomically positioned vertical slit-like meatus, a normal urinary stream, and a satisfactory cosmetic appearance based on the patients’ and parents’ perspectives.

### 2.3. Statistical Analysis

All numerical variables are presented by mean and standard deviation values, while all categorical variables are interpreted by ratios. The comparison of categorical variables was performed using the Chi-square test.

## 3. Results

In total, 104 patients were included in the study. Eighty (76.9%) patients presented with anterior penile hypospadias, while 24 patients (23.1%) had mid-penile hypospadias. The mean width of the glans was measured at 14.80 ± 0.96 mm, while in 16 patients (15.4%) testosterone was administered preoperatively. Chordee was detected in 9 patients (8.7%). More precisely, the mean width of the glans in the anterior and mid-penile groups was 14.82 ± 0.94 mm and 14.64 ± 1.10 mm, respectively. In the anterior hypospadias group, chordee was detected in 3 patients (3.8%), while preoperative testosterone administration was utilized in 12 patients (15%). In the mid-penile hypospadias group, chordee was detected in six patients (25%), while preoperative testosterone administration was utilized in four patients (16.67%) (Table 1).

**Figure 3:**
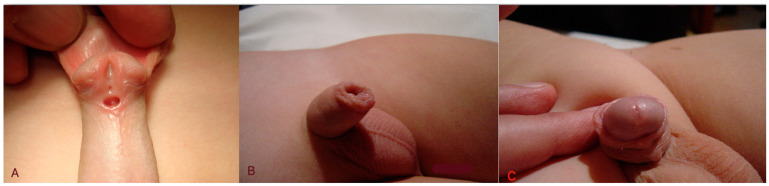
The pre- (**A**) and postoperative (**B**,**C**) appearance of the same case.

The mean age of the patients at the time of the surgery was 20.8 ± 12.4 months, while the mean operative time was 114.2 ± 28.7 min. The mean follow-up was 101.1 ± 44.8 months. All the operations were completed uneventfully, and all the patients were discharged on the first postoperative day. All the cases of chordee were resolved after the degloving of the penis, and no remaining chordee was observed during the follow-up. No early postoperative complications were detected. During the follow-up period, 16 (15.4%) urethral fistulas and one (0,9%) meatal stenosis were recorded, while no dehiscence was noticed. More precisely, in the anterior penile group, 12 (15%) urethral fistulas and one (1.3%) meatal stenosis were noted, while in the mid-penile hypospadias group, four (16.7%) urethral fistulas were noted (Figure 3) (Table 2). The patient suffering from meatal stenosis did not have a history of chordee or preoperative testosterone stimulation. Concerning postoperative fistulas, three (18.8%) and five (31.3%) patients had a history of chordee and preoperative testosterone stimulation, respectively. The differences were not statistically significant (*p* = 0.12 and *p* = 0.056, respectively).

## 4. Discussion

In this study, the 12-year experience in the management of distal hypospadias with 20-h catheterization was described. The mean age of the patients at the time of the surgery was 20.8 ± 12.4 months, and all the operations were completed uneventfully. The urethral catheter was removed 20 h postoperatively, and all patients were discharged on the first postoperative day. During the long follow-up period, which was 101.1 ± 44.8 months, complications were recorded in 17 (16.3%) patients, while the majority of them occurred in the group of anterior hypospadias.

The TIP urethroplasty was performed in all the patients of our study, as it constitutes one of the established surgical techniques independent of the chordee severity based on the EAU Guidelines [15]. Hamid et al. conducted a prospective study comparing the use of Buck’s Fascia or not in the hypospadias repair, performing TIP urethroplasty on 164 distal and mid-penile hypospadias [16]. The TIP technique was also utilized in 71.9% and 88.9% of patients who suffered from distal and mid-penile hypospadias, respectively, in a retrospective study conducted by Hild et al. [17]. Moreover, a systematic review and meta-analysis conducted by Wu et al. revealed no statistically significant difference concerning the complications between the TIP and other surgical techniques in non-proximal hypospadias [18].

Although the placement of a urethral catheter or stent in TIP urethroplasty constitutes a major factor in tissue healing, it has been associated with postoperative discomfort and meatal stenosis as well as bladder spasm and detrusor muscle contraction even when anticholinergic and antibiotic medication is administrated [7,9,10,19,20,21,22]. According to Snodgrass and Yucel’s recommendation, urethral stents should be placed for five to seven days to improve tissue healing and limit complication risk [23]. Additionally, the risk of urethrocutaneous fistula is minimized when the urethral catheter is placed for some days [19,23]. The literature data indicate that the time needed for partial epithelialization is five days, and for complete epithelialization and normal urothelium, two weeks [7,21]. In their study, Ning Xu et al. questioned the need for catheterization in boys with hypospadias treated with TIP urethroplasty and reported minimal complications and patient discomfort in non-catheter TIP [10]. Based on these findings, we assumed that removing the catheter the first day postoperatively could retain an equilibrium between the epithelialization process and the prevention of stenosis or strictures formation.

Although TIP urethroplasty is the treatment of choice worldwide in distal hypospadias repair, the use of a urethral catheter as well as the duration of its placement remains a matter of controversy. Some studies optimize the use of urethral catheters instead of stents regarding both efficient urinary drainage and safety [24,25]. In their comparative study, Arda and Mahmutoğlu compared the incidence of complications in hypospadias repair patients in two approaches, i.e., placement of a urethral catheter or placement of an 8Fr feeding tube as a stent [22]. The use of a urethral catheter was associated with a lower complication rate in stented patients [22]. In our study, a 6 Fr Foley 100% silicon urethral catheter was utilized as a postoperative drainage approach.

Regarding the appropriate duration of ureteral catheter placement, Kumar A et al. compared the outcomes of TIP urethroplasty following early versus late bladder catheter removal in 62 cases [7]. Early and late removal was defined as the removal of the catheter before and after the fifth postoperative day, respectively. All patients were evaluated two weeks, one month, three months, and six months postoperatively. Neither short-term (wound infection, urinary tract infection, urinary retention) nor long-term complication risk was affected by the early removal of the catheter [7]. However, in the group of early catheter removal, less patient discomfort was noted compared to the group of late catheter removal [7]. In contrast, Daher et al. reported better outcomes and significantly fewer complications with three-week instead of one-week catheterization at hypospadias repair with the Duplay technique [26]. In their retrospective study, Aslan et al. investigated the outcome of short-term catheterization in 128 children treated with TIP urethroplasty for distal hypospadias [27]. The urethral catheter was removed before and after 24 h in 99 and 29 patients, respectively. Although complications (fistula, meatal stenosis, tube dehiscence, and buried penis) occurred in 11.1% of the first group and 13.8% of the second one, no statistically significant difference was detected [27]. The outcomes of our study are comparable to those of Aslan et al. as the complication rate with 20-h postoperative catheterization was 16.3%.

The type of catheter placed in TIP urethroplasty in hypospadias patients is another issue to be addressed. The effectiveness of both silicone and feeding catheters in patients treated with TIP urethroplasty was evaluated in the retrospective study of Arpacik and Yildiz [28]. No statistically significant difference was observed in hypospadias-related complications. However, a higher incidence of catheter-related complications such as catheter occlusion, urine leak around the catheter, and spontaneous withdrawal was observed in patients with feeding catheters. A modified Foley catheter, i.e., a hole-end catheter, was proposed by Ghareeb and Azooz to provide better bladder drainage with the ability to easily dislodge precipitations [25]. Polat et al. proposed the use of a latex Foley catheter for 1–2 days as a safe approach with a minimum complication rate [29]. Additionally, in their patient series, the catheter was not sutured in the glans and the balloon was infiltrated with 2 mL. Finally, in their experimental study, Hosseinpour et al. compared the postoperative short-term clinicopathological complications of latex and silicone catheters in rabbits with hypospadias repair (TIP urethroplasty) [30]. The safety and feasibility of the less expensive latex catheters were reported, and these catheters were proposed by the authors for short-term postoperative use in patients without latex hypersensitivity.

Our study shows certain limitations. Although the follow-up period seems to be adequate for the extraction of the trend of the outcomes, the sample size is relatively small. The lack of a control group may also be considered as a limitation. Further studies comparing early and late catheter removal in patients operated by the same surgeon should be conducted. However, the aim of this study was to retrospectively analyze and interpret the long-term outcomes of TIP urethroplasty with 20-h catheterization.

## 5. Conclusions

In hypospadias repair with TIP urethroplasty, the application of 20-h catheterization seems to be a feasible alternative with outcomes comparable to other conventional drainage approaches and reduced discomfort for the patients without increased risk for long-term complications.

## Figures and Tables

**Table 1:** Preoperative data of the patients.

Variable	Outcome
Patients (n)	104
Anterior Penile Hypospadias (n, %)	80 (76.9%)
Mid-Penile Hypospadias (n, %)	24 (23.1%)
Glandular Width (mm, mean ± SD)	14.80 ± 0.96
Anterior Penile Hypospadias (mm, mean ± SD)	14.82 ± 0.94
Mid-Penile Hypospadias (mm, mean ± SD)	14.64 ± 1.10
Preoperative Testosterone (n,%)	16 (15.4%)
Anterior Penile Hypospadias (n, %)	12 (15%)
Mid-Penile Hypospadias (n, %)	4 (16.67%)
Chordee (n, %)	9 (8.7%)
Anterior Penile Hypospadias (n, %)	3 (3.8%)
Mid-Penile Hypospadias (n, %)	6 (25%)

**Table 2:** Intra- and Postoperative data of the patients.

Variable	Outcome
Age at operation (months, mean ± SD)	20.8 ± 12.4
Operative time (min, mean ± SD)	114.2 ± 28.7
Complications (n, %)	17 (16.3%)
Urethral Fistulas (n, %)	16 (15.4%)
Urethral Stenosis (n, %)	1 (0.9%)
Anterior Penile Hypospadias Complications (n, %)	13 (17.5%)
Urethral Fistulas (n, %)	12 (15%)
Urethral Stenosis (n, %)	1 (1.3%)
Mid-Penile Hypospadias Complications (n, %)	4 (16.7%)
Urethral Fistulas (n, %)	4 (16.7%)
Urethral Stenosis (n, %)	0 (0%)

## Data Availability

The data that support the findings of this study are available from the corresponding author, upon reasonable request.
